# Power-free plasma separation based on negative magnetophoresis for rapid biochemical analysis

**DOI:** 10.1038/s41378-024-00837-8

**Published:** 2024-12-31

**Authors:** Lin Zeng, Chao Liu, Yi Yang, Shi Hu, Ruihan Li, Xiaotian Tan, Jienan Shen, Yi Zhang, Shaohui Huang, Hui Yang

**Affiliations:** 1 Research Center for Bionic Sensing and Intelligence, Institute of Biomedical and Health Engineering, Shenzhen Institutes of Advanced Technology, Chinese Academy of Sciences, 518055 Shenzhen, China; 2 Marine Engineering College, Dalian Maritime University, 116026 Dalian, China; 3 Research Center for Medical AI, Institute of Biomedical and Health Engineering, Shenzhen Institutes of Advanced Technology, Chinese Academy of Sciences, 518055 Shenzhen, China; 4 School of Biosciences, University of Chinese Academy of Sciences, 101408 Beijing, China

**Keywords:** Engineering, Chemistry

## Abstract

We present a versatile platform for label-free magnetic separation of plasma, tailored to accommodate diverse environments. This innovative device utilizes an advanced long-short alternating double Halbach magnetic array, specifically engineered for optimal magnetic separation. The array’s adaptability allows for seamless integration with separation channels of varying sizes, enabling static separation of whole blood. The platform has a highly flexible processing throughput, spanning from 100 μL to 3 mL per separation cycle without sacrificing separation efficiency. A key aspect of this device is its power-free operation throughout the separation process, obviating the complexity of conventional separation devices. Its effectiveness is demonstrated by the extraction of 40 μL of plasma from 100 μL of rat whole blood within 8 min. The separated plasma proved effective for subsequent analysis of antibody concentration and size in the separated plasma for pharmacokinetic investigations, yielding results on par with those obtained via centrifugation. Furthermore, the device’s high-throughput capability was validated using human whole blood, achieving 3 mL of plasma separation in just 1 min. In a follow-up study on COVID-19 IgG antibody detection, the results matched those from centrifugation. The device demonstrates a separation efficiency of 99.9% for cells larger than 1 μm in both rat and human blood samples, with a plasma recovery rate of 72.7%. In summary, our magnetic separation device facilitates rapid plasma extraction from whole blood, with a capacity of up to 3 mL per minute in human blood, without compromising subsequent plasma-based analyses, thereby highlighting its broad applicability across diverse settings.

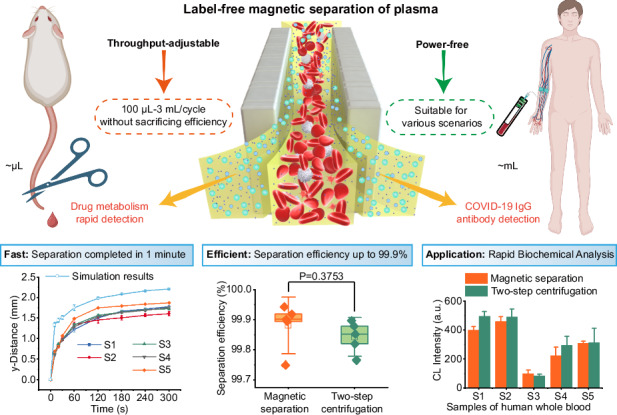

## Introduction

The pretreatment of whole blood plays a pivotal role in ensuring the accuracy of subsequent biochemical detection and analysis. Notably, a significant proportion (80%) of errors in laboratory medicine originates “outside the laboratory walls”^1^. Given the potential interference of blood cells with downstream detection processes, the extraction of plasma through centrifugation plays a crucial role in the initial plasma extraction step for most diagnostic assays^2^. This is particularly true in routine blood biochemical testing, trace biomarker analysis, and multiple marker testing, where centrifugation method becomes imperative to yield substantial volumes of undiluted plasma within a specific timeframe. Currently, centrifuges present as the most efficient and widely employed equipment for plasma extraction during these procedures.

However, with the advent of point-of-care testing (POCT), blood-based testing often requires only small volumes of plasma sample (μL)^3,4^. In such scenarios, centrifugation proves suboptimal, particularly due to challenges in integrating it with POCT equipment. Therefore, various methods tailored for micro-volume plasma separation, such as microfluidics^5–7^, have been developed. Moreover, in specialized settings like wilderness environments, emergency scenes, and regions with limited power supply, traditional centrifuges are considered impractical luxuries. In these contexts, there is a pressing need for simple power-free devices capable of rapidly extracting plasma to facilitate subsequent testing^8–12^. In addition, in the realm of drug delivery, release, and metabolism studies in animals, rapid separation of small blood samples in animal models is imperative for detecting critical information like drug concentrations^13,14^. This further underscores the necessity for alternative rapid plasma separation methods, as centrifuges may not be conducive in terms of throughput and time efficiency.

Due to centrifuges’ limitations in handling trace samples and stringent environmental prerequisites, alternative plasma extraction methods have emerged in recent years. These methods can generally be categorized into active and passive separation techniques. Active methods primarily rely on phenomena like acoustics, electricity, and magnetism to actively exclude or capture blood cells. For example, Rasheed et al.^15^ and Huang et al.^16^ utilized sound waves with microfluidic chips, while Habibiyan et al.^17^ employed electrophoretic force and gravity effects. Fan et al.^18^ harnessed dielectric forces, and Erickson et al.^19^ used a power-free device with magnetic beads and permanent magnets to enrich red blood cells in situ via magnetophoresis, although this approach can process 1 mL of whole blood samples, it necessitates the magnetic labeling of red blood cells and proves ineffective for other blood cell types. Conversely, Kang et al.^20^ introduced nanomagnetic particles into whole blood and employed the negative magnetophoresis effect generated by a permanent magnet array to separate blood cells, with the flow rate at the outlets precisely regulated by a micropump to selectively retain or remove platelets. Nevertheless, the throughput of this method remains limited; in a large channel with a cross-sectional area of 80 mm^2^, the throughput could only reach 100 μL/min. While active separation methods typically boast high extraction efficiency and purity, they are not without their drawbacks. These methodologies require the generation of corresponding sound fields, electric fields, and magnetic fields, and rely on high-precision pumps for accurate flow rate control in continuous flow separation. This inherent complexity poses challenges in integrating these methods into POCT equipment and renders them unsuitable for deployment in specialized scenarios as aforementioned.

Passive separation methods, on the other hand, commonly involve filter membranes for blood cell filtration, encompassing conventional^21^ and microfluidics-based approaches^22–24^. These methods, including membrane filtration integrated with capillary effects^25,26^, are relatively straightforward to operate and seamlessly interface with detection systems. However, challenges such as sample loss, clogging, limited processing rates that hinder their suitability for trace analysis with large sample volumes, and necessity of blood dilution in many instances. In microfluidics, fluid dynamics like inertia and lateral flow are leveraged to separate blood cells^27–30^, but they have stringent flow rates and channel structure requirements, limiting their applicability. Nonetheless, innovative passive separation methods like the centrifugal-based fidget-spinner^31^ and paper centrifuge^32^ enable rapid separation of trace blood samples within power-free devices.

Reflecting on the current research landscape, it becomes evident that different application scenarios and detection targets necessitate distinct processing methods for both small and large volume whole blood samples. The lack of a unified and effective method adaptable to different scenarios and blood sample volumes remains a challenge. Moreover, apart from a few manual separation techniques, most methods rely on precision pumps for flow rate control, posing obstacles for power-free device development.

To address these challenges, we propose a power-free device based on the principle of negative magnetophoresis. As one of the magnetic separation methods, negative magnetophoresis, unlike magnetophoresis which necessitates magnetic bead labeling, is distinguished by its capacity to sort target particles by size in a label-free manner. Following the advent of biocompatible ferrofluids, negative magnetophoresis has progressively been employed for cell manipulation and sorting. Examples include the utilization of microfluidic chips to achieve continuous separation of HeLa cells and blood cells^33^, the isolation of rare cells such as circulating tumor cells^34,35^, and even the extraction of nanoscale extracellular vesicles^36,37^. Compare to the traditional centrifugation and emerging microfluidic technology, the power-free magnetic separation device is applicable across all scenarios and has no throughput limitation. Most importantly, it can efficiently separate plasma from almost any volume of human whole blood within just 1 min. We validate the system’s separation capability and broad applicability through experiments using rat and human whole blood, along with subsequent plasma marker detection experiments.

## Results

### System design

**Fig. 1:**
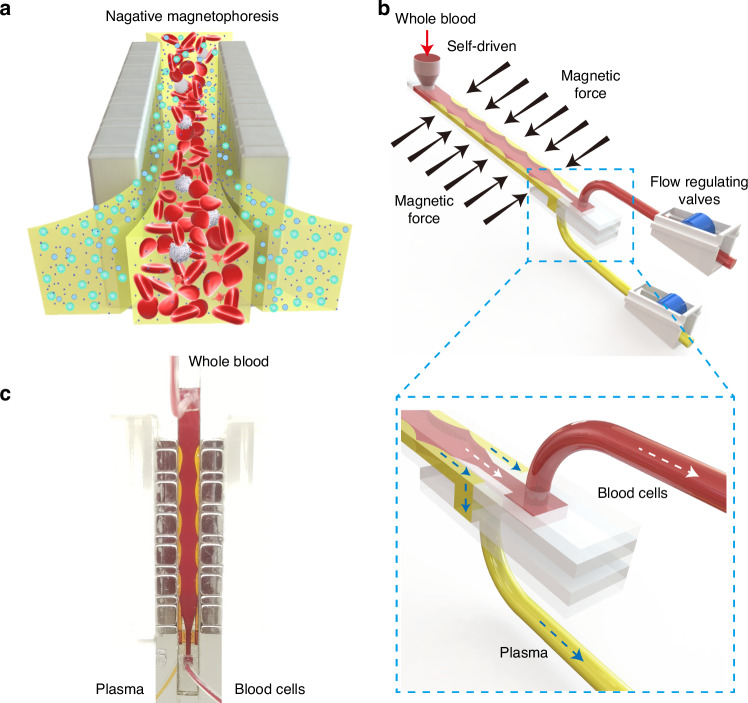
**Schematic diagram of the self-driven negative magnetophoresis separation system incorporating an enhanced alternating double Halbach magnetic array**. **a** Illustration on the principle of negative magnetophoresis separation, highlighting the repulsion of non-magnetic particles from the magnetic field. **b** Schematic diagram of the entire separation system, depicting the integration of the enhanced magnetic array with the separation channel. **c** Photo of the separation area, providing a visual representation of the experimental setup

The proposed negative magnetophoresis separation system is shown in Fig. 1a. As a mixture of whole blood and ferrofluid (a colloidal suspension of magnetic nanoparticles) enters the magnetic field zone, the ferrofluid undergoes concentration gradient formation due to its attraction to the high magnetic field gradient^38^. This gradient results in a concentrated region of magnetic fluid proximal to the magnet and a lower concentration further away. Consequently, this concentration gradient facilitates the segregation of whole blood components within the magnetic field, where non-magnetic particles are repelled from the magnet, exhibiting the negative magnetophoretic effect. The degree of repulsion is proportional to particle size, larger blood cells are displaced towards the channel’s centerline, while plasma tends to accumulate on both sides of the channel near the magnet array.

To enhance separation efficiency, an innovative design featuring an enhanced long-short alternating double Halbach magnetic array is introduced. This array comprises magnets with dimensions of 5 mm × 5 mm × 20 mm and 5 mm × 2 mm × 20 mm, arranged alternately. Central to this design is the integration of a throughput-adjustable separation channel (with adjustable height H and length L, while maintaining a fixed width W of 5 mm) within the enhanced magnetic array structure. The detailed design of the separation channel is shown in Supplementary Fig. S1. The channel, constructed from cost-effective materials such as polyethylene terephthalate (PET) or via plastic 3D printing (as shown in Supplementary Fig. S2), allows for precise regulation of the throughput based on its dimensions. The enhanced magnetic array generates a high-intensity negative magnetophoretic force across a wide range within the separation channel, enabling efficient blood cell separation and plasma extraction. Gravity-driven flow directs whole blood into the separation area, where plasma separation occurs. At the channel’s exit, a double-layer chip separates plasma from blood cells (Fig. 1b), with flow rates regulated by manual flow control valves. A photograph of the separation area is shown in Fig. 1c.

### Theoretical analysis

#### Magnetic field and negative magnetophoretic force

The negative magnetophoretic force experienced by particles (approximated as spherical) within the magnetic field is given by:

1 $${{\boldsymbol{F}}}_{m}=\frac{{\rm{\pi }}{d}_{p}^{3}\nabla \chi }{6{\mu }_{0}}\left({\boldsymbol{B}}\cdot {\boldsymbol{\nabla }}\right){\boldsymbol{B}}{=}\frac{{\mu }_{0}{\rm{\pi }}{d}_{p}^{3}}{6}\left[\left({{\boldsymbol{M}}}_{p}{\boldsymbol{-}}{{\boldsymbol{M}}}_{f}\right){{\cdot}}{\boldsymbol{\nabla }}\right]{\boldsymbol{H}}$$

Where *d*_*p*_ is the diameter of the non-magnetic particle, $\nabla \chi$ is the difference in magnetic susceptibility between the particle and the surrounding ferrofluid, ***B*** is the magnetic induction intensity, *μ*_0_ is the vacuum permeability, ***M***_*p*_ is the magnetization of non-magnetic particles, and ***M***_*f*_ is the local ferrofluid magnetization exposed to an external magnetic field ***H***, which is given by:

2 $${{\boldsymbol{M}}}_{f}={C\left({\boldsymbol{r}}\right){\boldsymbol{M}}}_{{np}}$$

Where ***M***_*np*_ is the magnetization of magnetic nanoparticles suspended in the ferrofluid, with its magnitude *M*_*np*_ given by ref. ^39^:

3 $${M}_{{np}}{=}{M}_{s}L\left(\xi \right){=}{M}_{s}\left[\coth \left(\xi \right)-{\xi }^{-1}\right]$$

The ratio of local magnetostatic energy *ξ* is given by:

4 $$\xi =\frac{{{\mu }_{0}{\rm{\pi }}{d}_{{np}}^{3}M}_{s}H}{6{k}_{B}T}$$

Where *M*_*s*_ and *d*_*np*_ represent the saturation magnetization and the diameter of the magnetic nanoparticle suspended in the ferrofluid, respectively. *k*_*B*_ and *T* represent Boltzmann’s constant and the absolute temperature, and *H* is the magnetic field at the local position.

The local volume fraction of magnetic nanoparticles *C*_*m*_(***r***) in the gradient magnetic field is calculated by ref. ^38^:

5 $${C}_{m}\left({\boldsymbol{r}}\right)={\left[1+\frac{1-{\Phi }_{m}}{{\Phi }_{m}}\frac{\sinh ({\xi }_{0})}{\sinh (\xi )}\frac{\xi }{{\xi }_{0}}\right]}^{-1}$$

**Fig. 2:**
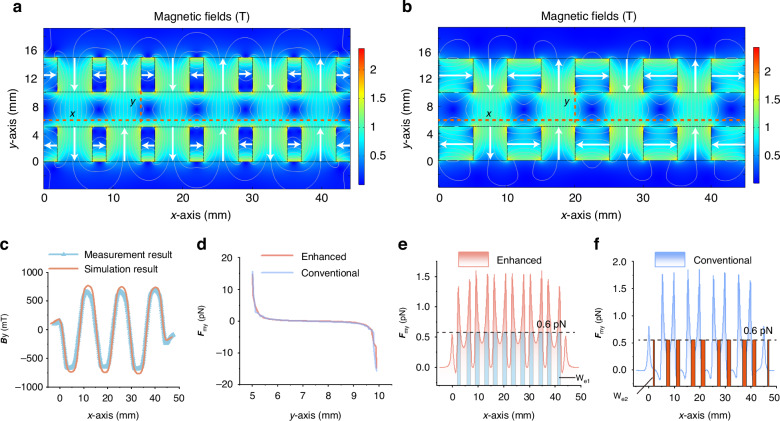
**Schematic illustration of magnetic field simulation results and its enhancement mechanism**. **a** Simulation results of the enhanced double Halbach magnetic array, showing the magnetic field distribution with white arrows indicating the magnetic field direction. **b** Comparable simulation results for the conventional double Halbach magnetic array. **c** Comparative analysis of simulation results and experimental measurements for the enhanced double Halbach magnetic array, validating the accuracy of the simulation model. **d** Comparison of the negative magnetophoretic force exerted on 2 μm particles along the y-axis by both enhanced (at x = 14 mm) and conventional (at x = 20 mm) magnetic array, as indicated by the dotted lines in Fig. 2a, b. **e**, **f** The negative magnetophoretic force profiles on 2 μm particles along the x-axis (at y = 6 mm) for the enhanced and conventional magnetic arrays, respectively, highlighting the effective length of the negative magnetophoretic force generated by each array

Where Φ_*m*_ is the concentration of magnetic nanoparticles in the bulk fluid far from the magnetic field, and *ξ*_0_ is the ratio for a nanoparticle in the bulk fluid far from any sources of local magnetic field. Based on the above equations and the properties of the ferrofluids^40^, the concentration distribution along the y-axis (Fig. 2a) is shown in Fig. S3.

From Eq. (1), it becomes evident that, in addition to particles and medium parameters, the negative magnetophoretic force is proportional to the product of magnetic field intensity and gradient. To validate the enhanced magnetic array’s performance, simulation analyses comparing the enhanced and conventional double Halbach magnetic arrays were conducted (Fig. 2a, b, respectively). Magnetic field scans (along the x-axis, 1 mm from the magnet array) verified the simulation’s accuracy (Fig. 2c). Further, the negative magnetophoretic forces generated by both array on 2 μm particles were compared at a distance of 1 mm along the x-axis (Fig. 2d). Previous studies have shown that the maximum negative magnetophoretic force required to drive 2 μm particles is approximately 0.6 pN^41,42^. Fig. 2d demonstrates that both arrays generate sufficient forces to drive 2 μm particles. Consequently, the effective action range (or time) of the negative magnetophoretic force emerges as a pivotal factor in enhancing separation efficiency.

To analyze this range, the negative magnetophoretic force ***F***_*my*_ (y-component) exerted on 2 μm particles was simulated along both the y-axis and x-axis directions. Along the y-axis, ***F***_*my*_ generated by the enhanced and conventional magnetic arrays are comparable. However, significant disparities were observed along the x-axis (Fig. 2e, f). Setting 0.6 pN as the threshold, the length along the x-axis where ***F***_*my*_ exceeds this value was defined as the effective length W. The enhanced magnetic array exhibited an effective length of W_e1_ = 17.86 mm, while the conventional magnetic array had W_e2_ = 11.93 mm, presenting a 49.7% increase in the x-axis action range (1 mm from the magnet edge). This enhancement underscores the significant efficiency improvement offered by the enhanced magnetic array within a comparable size range as its conventional counterpart.

#### Particle motion simulation and optimization

In addition to the negative magnetophoretic force, particles are also affected by hydrodynamic drag, gravity, and buoyancy forces within the channel. Gravity and buoyancy largely cancel each other out, simplifying considerations to only the first two forces. The hydrodynamic drag force ***F***_*d*_ is given by:

6 $${{\boldsymbol{F}}}_{d}=3\pi {\eta }_{f}{d}_{p}{f}_{d}\left({{\boldsymbol{u}}}_{f}-{{\boldsymbol{u}}}_{p}\right)$$

where *η*_*f*_ is the ferrofluid viscosity, *f*_*d*_ is the particle drag coefficient, ***u***_*f*_ and ***u***_*p*_ are the velocity vectors of the ferrofluid and particles, respectively. Therefore, the particle’s equation of motion is:

7 $${m}_{p}\frac{d{{\boldsymbol{u}}}_{p}}{{dt}}={{\boldsymbol{F}}}_{m}+{{\boldsymbol{F}}}_{d}$$

Treating the dilute magnetic fluid as an incompressible Newtonian fluid, the steady-state flow within the channel is governed by mass conservation and momentum conservation equations:

8 $$\nabla \cdot {{\boldsymbol{u}}}_{{\boldsymbol{f}}}=0$$

9 $${\rho }_{f}{{\boldsymbol{u}}}_{{\boldsymbol{f}}}\,{{\cdot}}\,\nabla {{\boldsymbol{u}}}_{{\boldsymbol{f}}}=-\nabla p+\eta {\nabla }^{2}{{\boldsymbol{u}}}_{{\boldsymbol{f}}}$$

**Fig. 3:**
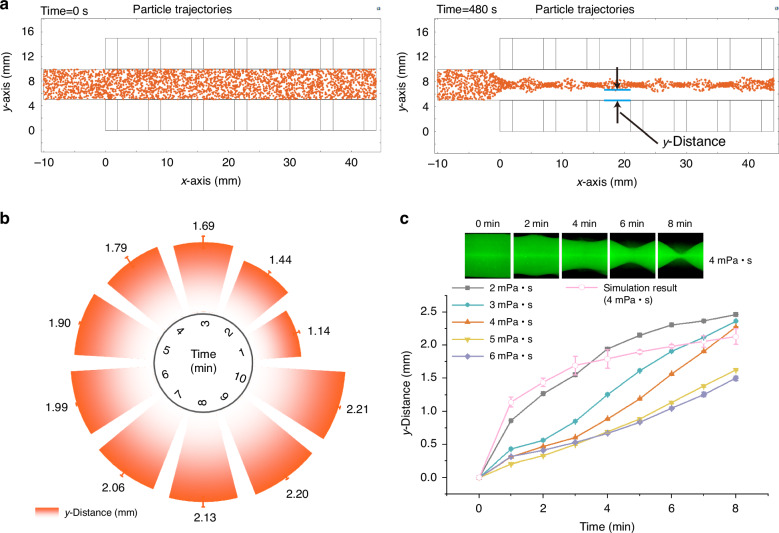
**Simulation of particle motion and optimization of experimental parameters**. **a** Trajectories of 2 μm particles within the separation channel at the initial time (0 min) and after 8 min; **b** Correlation between y-distance and time based on simulation data; **c** Relationship between y-distance and time observed experimentally across various viscosities, and comparison between simulation results and experimental results

**Fig. 4:**
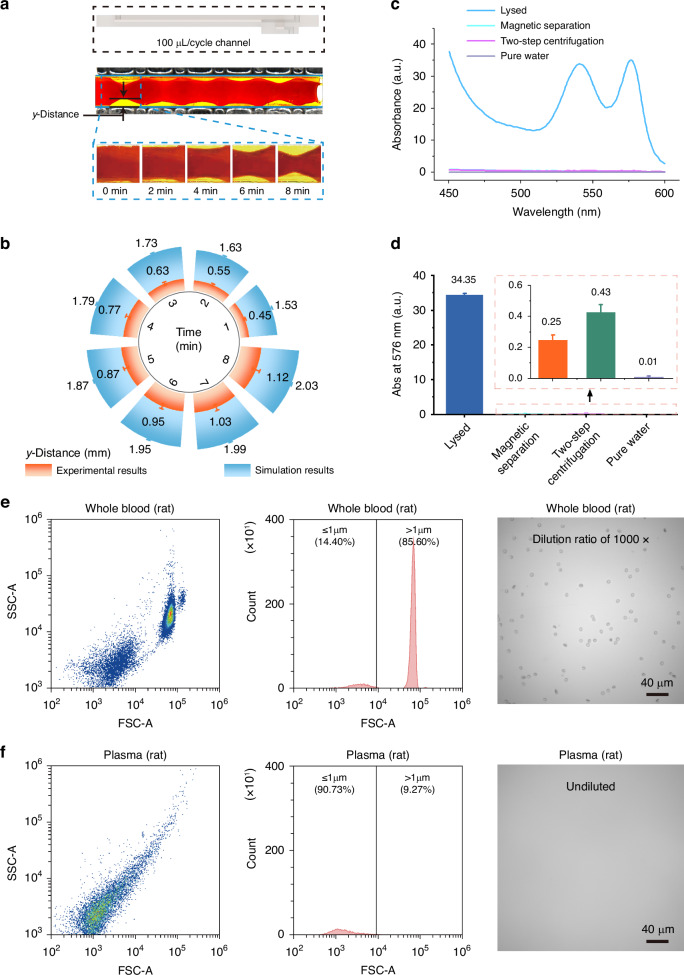
**Separation results of rat whole blood**. **a** Photographs of whole blood separation using a 100 µl/cycle channel; **b** Correlation between y-distance and time during the separation of rat plasma, and comparison between simulation results (4 μm particles) and experimental results; Hemolysis test results, where (**c**) shows the OD spectra of various samples, and (**d**) provides a quantitative comparison of OD at 576 nm for these samples; **e** Flow cytometry results and microscopic image of blood cells in the whole blood sample; **f** Flow cytometry results and microscopic image of blood cells present in the plasma after separation

By solving the particle motion equation and flow field equations, particle trajectories within the channel were obtained, as shown in Fig. 3. Static separation simulations reveal complete repulsion of 2 μm particles by the magnetic array, focusing them at the channel’s centerline within 8 min (Fig. 3a). Tracking particle movement over time along the y-distance revealed an initial rapid pace in the first 3 min, followed by deceleration (Fig. 3b). This deceleration is attributed to diminishing magnetic force as particles migrate towards the center, as well as increasing particle concentration which impedes further movement. The viscosity of the ferrofluid used for simulation here is 4 mPa • s, which is similar to whole blood. Since the viscosity directly affects the hydrodynamic drag force ***F***_*d*_, experimental verifications using 2 μm fluorescent particles in ferrofluids with varying viscosities confirmed particle migration beyond 1 mm from the magnet edge within 6 min, regardless of viscosity conditions (Fig. 3c). These findings guide subsequent parameter selection for real blood sample separation. In the following experiments, the separation cutoff point (*w*) was chosen as 1 mm from the last main magnet’s edge, as shown in Supplementary Fig. S1. Furthermore, we used 4 μm particles and 7 μm particles to simulate rat blood cells and human blood cells, respectively, the simulation results are shown in Figs. 4b and 6b.

### Performance verification using rat whole blood separation

To validate the system’s separation capabilities, a plasma separation experiment using rat whole blood was conducted, as shown in Fig. 4. With channel dimensions of 0.5 mm height and 39.5 mm length, a processing throughput of approximately 100 μL/cycle was achieved (Fig. 4a). The relationship between plasma separation distance and time is shown in Fig. 4b. To maximize the removal of blood cells from the plasma, we optimized the separation duration to 8 min (Fig. 4b and Movie S1), that is, we opened the flow regulating valve at the rear end after 8 min, and guided the plasma and blood cells to the corresponding outlets respectively, the plasma recovery rate reached 72.7%.

We investigated the impact of our separation protocol on hemolysis during blood cell separation, as shown in Fig. 4c, d. Here, optical density (OD) spectral measurements were performed on plasma samples obtained via our negative magnetophoresis method, plasma samples collected through centrifugation, and plasma samples derived from centrifuging whole blood after adding hemolyzing agent. The results showed that plasma separated using our method exhibited similar characteristics to plasma separated by centrifugation. Furthermore, in comparison to plasma separated after the addition of a hemolyzing agent (positive control), our method induced minimal hemolysis during the separation of whole blood. To substantiate the effectiveness of blood cell removal via negative magnetophoresis, we employed flow cytometry to quantify whole blood samples prior to separation and plasma samples following separation. The results, shown in Fig. 4e, f, indicate that in whole blood, cells larger than 1 μm constituted 85.60% of the total, whereas only 9.27% remained in the plasma after separation. This signifies a blood cell removal rate of 99.9%.

### Application in simulated drug metabolism rapid detection

**Fig. 5:**
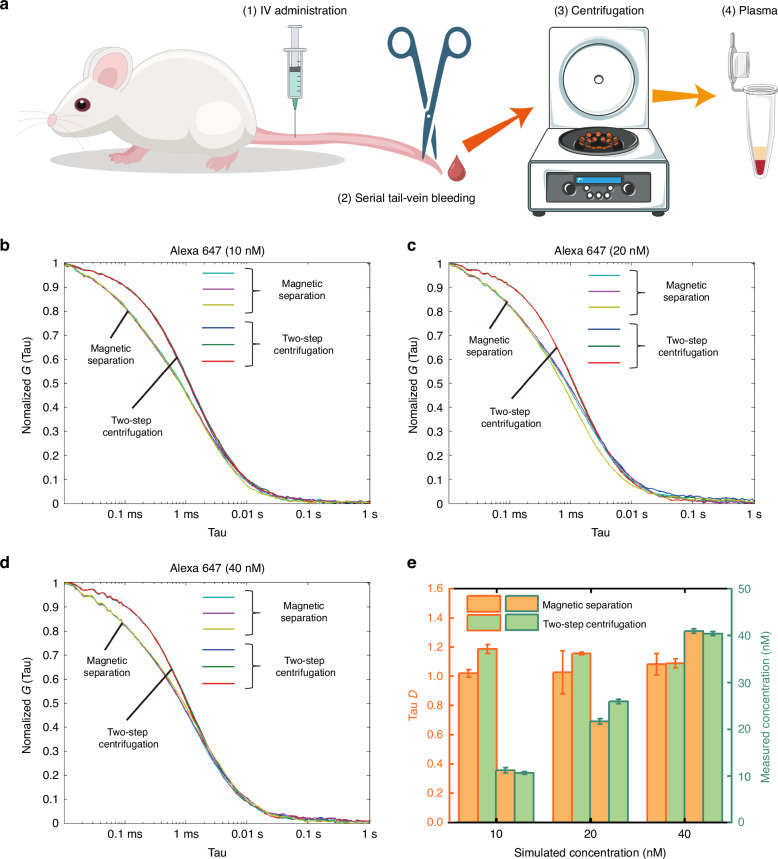
**Simulated drug metabolism rapid detection results**. **a** The process of drug metabolism detection in rats; **b**–**d** FCS test results for fluorescent antibodies in plasma, isolated using magnetic separation and centrifugation. The concentrations of the antibodies are (**b**) 10 nM, (**c**) 20 nM and (**d**) 40 nM, respectively; **e** Comparation on the fluorescent antibody concentration and Tao D value (a measure related to antibody size) in plasma samples processed via magnetic separation and centrifugation

In pharmacokinetic studies involving rats/mice, it is essential to collect microvolume (~100–200 μl) whole blood every 5–15 min and extract plasma for analysis^13^, the process is shown in Fig. 5a. However, centrifugation for such samples and processing frequency is challenging and prone to errors. As an alternative, the proposed negative magnetophoresis method efficiently separate plasma from trace whole blood samples (100 μL) in a short period, as demonstrated above. To assess its efficacy, fluorescent antibodies mimicking antibody drugs were mixed with rat whole blood, separated to obtain plasma, and analyzed using fluorescence correlation spectroscopy (FCS). Compared to centrifugation controls (Fig. 5b–d), the negative magnetophoresis method closely matched Tau D values (concerning parameters related to the particle size of fluorescent antibodies) and concentrations obtained through centrifugation (Fig. 5e). Measurement variations were within acceptable ranges, which is 14.1%, 11.3% and 0.6% for Tau D and 5.6%, 16.3% and 1.4% for concentration of three experiment groups.

### Human whole blood separation and COVID-19 IgG antibody detection

**Fig. 6:**
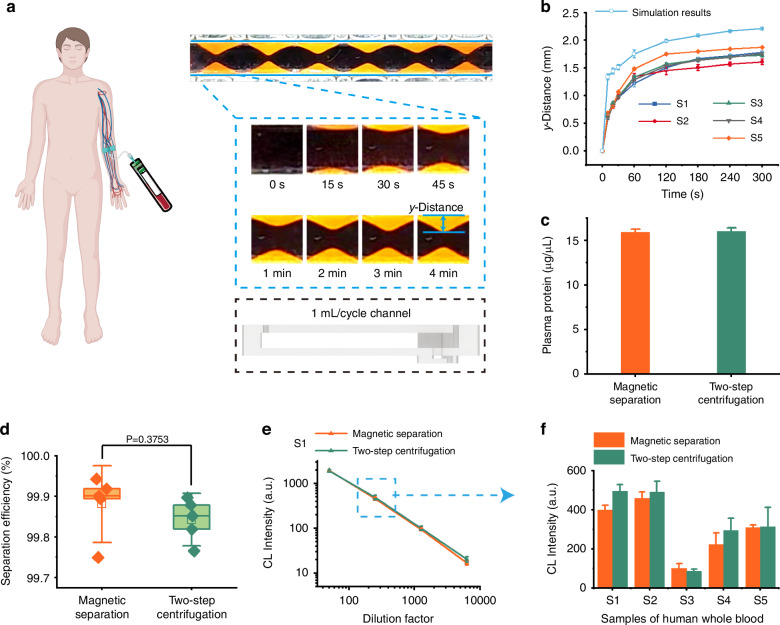
**Human plasma separation and COVID-19 IgG antibody test results**. **a** Photographs of whole blood separation using a 1 ml/cycle channel; **b** Relationship between y-distance and time during the separation of human plasma obtained from 5 volunteers, and comparison between simulation results (7 μm particles) and experimental results; **c** Determination results of total protein content in human plasma obtained by magnetic separation and centrifugation; **d** Comparison of the separation efficiency between magnetic separation and centrifugation for 5 human whole blood samples; **e** SARS-CoV-2 S-ECD trimer IgG titration curve of human plasma (S1) obtained by the magnetic separation system and centrifugation; **f** SARS-CoV-2 S-ECD trimer IgG detection results of 5 human blood samples at the dilution factor of 250

The system’s capabilities are further extended to large-volume plasma separation from human whole blood. By adjusting channel height, throughputs of up to 3 mL/cycle were achieved. In order to better observe the separation effect, we used 1 mL/cycle channels to study the relationship between plasma separation distance and time, as shown in Fig. 6a, b and Movie S2. Experiments using human whole blood from 5 volunteers reveal significantly shorter separation times compared to rat blood, attributed to the larger size of human blood cells, blood cells can move to a position 1 mm from the edge of the magnet in 30 s. To ensure the extraction of purer plasma, we opted for a separation time of 60 s each cycle, and the separation process using 3 mL/cycle channel was shown in Supplementary Fig. S4.

Furthermore, we quantified the total protein content of plasma samples obtained through both negative magnetophoresis and centrifugation technique (Fig. 6c and Supplementary Fig. S5). The results demonstrate that the plasma protein concentration derived from negative magnetophoresis is comparable to that of centrifugation, suggesting that our method does not induce protein depletion. Subsequently, we employed flow cytometry to enumerate cells in the plasma (Supplementary Fig. S6), revealing that both methods effectively eliminated cells exceeding 1 μm in size (Supplementary. S7). Specifically, the magnetic separation achieved a separation efficiency of 99.9% (Fig. 6d), and the separation efficiencies of both methods were nearly identical across all groups (Supplementary Fig. S8). Finally, COVID-19 IgG antibodies were detected in the separated plasma using a tip-adapted optofluidic capillary immunoassay. The detection results obtained via negative magnetophoresis were highly concordant with those of centrifugation across all dilution factors (Fig. 6e, f and Supplementary Fig. S9). Therefore, plasma isolated using our negative magnetophoresis method is suitable for subsequent optical detection without compromising its quality or integrity.

## Discussions

This paper introduces an innovative, cost-effective, and power-free device for whole blood separation based on negative magnetophoresis. The device incorporates an enhanced long-short alternating double Halbach magnetic array in conjunction with biocompatible magnetic fluid, enabling efficient separation of blood cells from whole blood and subsequent plasma extraction. Notably, the separation effectiveness of this approach remains invariant to the channel dimensions, offering flexibility in achieving a broad range of whole blood processing throughputs, spanning from 100 μL/cycle to 3 mL/cycle, through adjustments in channel heights. Furthermore, the separation time remains consistent, ranging from 1 to 8 min per operation, depending primarily on blood type and viscosity. Notably, the separation rate achieved in this study for human whole blood reaches 3 mL/min, with potential for further enhancement by optimizing the magnetic array and separation channel dimensions.

A pivotal advantage of this approach lies in its reliance on static separation, thereby eliminating the need for driving pumps and enabling fully manual operation. This feature renders the proposed separation method highly versatile, making it suitable for diverse applications ranging from laboratory settings and routine inspections to POCT, remote environments, and resource-constrained areas.

To validate the efficacy of this system in plasma separation, experiments were conducted involving microvolume (100 μl) rat whole blood separation and antibody drug metabolism detection using fluorescent antibodies in the plasma. The results demonstrate that the separated plasma exhibits similar particle size and concentration to centrifugation-derived plasma, satisfying the requirements for rapid sorting of small sample volumes due to its high sorting efficiency. Additionally, the system’s capability for large volume (3 mL) separation was verified using 5 groups of human whole blood. Subsequent ELISA testing revealed effective detection of SARS-CoV-2 S-ECD trimer IgG in the plasma, with test results highly consistent with those obtained using centrifugation-derived plasma. Both rat and human blood separation achieved cell separation efficiencies of 99.9%, and the plasma recovery rate reached 72.7%.

A comprehensive comparison with existing literature reports reveals that the magnetic separation device presented in this paper exhibits significant advantages in terms of throughput (and its adjustment range) and separation rate, as detailed in Supplementary Table S1 and Fig. S10. These findings confirm that the system not only efficiently separates plasma from whole blood but also ensures that the separation process does not compromise the accuracy of downstream plasma detection, thus highlighting its potential for widespread adoption and impact in various applications.

## Materials and methods

### Fabrication of the system

The proposed system comprises magnetic arrays, each consisting of 6 main magnets and 7 auxiliary magnets, arranged in accordance with the magnetic field orientation depicted in Fig. 2. The magnets are securely affixed within a magnet holder using a robust adhesive. The distance between adjacent magnetic arrays is 5 mm. The dimensions of the main and auxiliary magnet are 5 mm × 5 mm × 20 mm and 5 mm × 2 mm × 20 mm respectively, with each magnetic array spanning a length of 44 mm. The center of the double magnetic array forms the separation channel, as illustrated in Supplementary Fig. S1.

The separation channel is stratified into upper and lower layers. The upper layer incorporates a sample inlet, a blood cell outlet, and a separation structure, designed to direct blood cells and plasma into distinct outlets. The lower layer serves as the plasma outlet. The width of the separation channel is set as 5 mm.

For microvolume separation, PET is utilized to fabricate the channel using an adhesive method. Specifically, laser cutting is employed to shape each layer, which is subsequently assembled using double-sided tape to form the separation channel. For large volume separations, 3D printing techniques are employed. To maximize the proximity of whole blood samples to the permanent magnet (thereby enhancing separation magnetic force), the channel sidewalls are uniformly constructed from 0.05 mm thick PET and affixed to the channel’s lateral surfaces using an adhesive. The sample inlet is equipped with a liquid reservoir for sample injection, while flow regulating valves are installed at the outlets to control sample flow within the channel.

### Details on the simulation

The separation model was built in COMSOL Multiphysics based on the channel design. The simulation employed the “Magnetic fields, No Current” module for magnetic field calculations, the “Laminar Flow” module for flow field computations, the “Particle Tracing for Fluid Flow” module for tracking particle trajectories in ferrofluid, and the “Fluid-Particle Interaction” feature in Multiphysics for multiphasic coupling. The parameters used are listed in Table S2.

### Preparation of whole blood samples

Ethylenediaminetetraacetic acid (EDTA)-treated whole blood samples were collected from Sprague-Dawley rats (8–10 weeks old) and tested under IRB (SIAT-IACUC-20230918-YGS-ZHZX-ZT-ZT-A0899-04) approved by Shenzhen Institute of Advanced Technology, Chinese Academy of Science. The samples were used immediately after collection.

Five groups of venous blood samples from healthy volunteers were collected in the EDTA-treated tube and tested under IRB (SIAT-IRB-230715-H0667) approved by Shenzhen Institute of Advanced Technology, Chinese Academy of Science. The samples were used immediately after collection.

### Experimental setup and separation experiments

Prior to separation, the whole blood sample was mixed with a biocompatible ferrofluid (Ferraheme, a US Food and Drug Administration-approved intravenous iron preparation from AMAG Pharmaceuticals, MA, USA) to achieve a volume concentration of 2% ferrofluid within the sample. The mixed sample was then injected into the injection reservoir, with the separation channel positioned vertically via a fixed bracket, ensuring the inlet positioned at the top and outlets at the bottom. The flow regulating values at the outlets are initially adjusted to facilitate sample entry and filling of the separation channel, following which the valves were closed to enable sample separation under static conditions. Upon completion of separation, the flow valve was reopened to direct plasma and blood cells into their respective outlets, thus concluding the plasma collection process.

If necessary, magnetic nanoparticles in the ferrofluid can be removed from the plasma using a syringe and a “magnetic filter” (a tube densely packed with 3–5 μm iron oxide microparticles), following a method outlined in a previous study^20^, the OD spectrum results of the plasma after removing the ferrofluid and the centrifuged plasma have highly consistent spectral curves, as shown in Supplementary Fig. S11. However, it is noteworthy that trace amounts of biocompatible magnetic nanoparticles have negligible impact on subsequent detection processes (Figs. 5e, 6e, 6f and Fig. S9). Therefore, magnetic nanoparticles were not removed from the samples in this study. In our future work, we can optimize and address this issue from the following three perspectives: (1) If the magnetic nanoparticles exert no influence on the subsequent detection, their removal becomes unnecessary. As an illustration, the two detection experiments conducted in this study did not involve the removal of magnetic nanoparticles. Furthermore, various biocompatible ferrofluid products or solutions are already available, which exert minimal impact on biological samples. (2) When magnetic nanoparticles necessitate removal, opting for a ferrofluid with slightly diminished dispersion stability can be advantageous, as it facilitates magnetic attraction and aggregation. This choice is predicated on the condition that conventional permanent magnets do not induce their aggregation and settlement. (3) An engineering solution for mass production of magnetic filter columns can be developed, enabling batch filling and allowing multiple filter columns to work simultaneously. This approach will enhance filtration throughput while minimizing the laborious steps associated with manual processing.

For comparison, two-step centrifugation control experiments were performed by centrifuging whole blood samples at ×1300 *g* for 10 min at 4 °C, followed by centrifugation at ×4000 *g* for 15 min at 4 °C to obtain platelet-free plasma^43^.

Microscopic imaging of blood cells was performed using a Zeiss Axio Observer 7 inverted microscope (Carl Zeiss Microscopy GmbH) equipped with an sCMOS camera (ORCA-Flash4.0-V3, Hamamatsu).

The separation efficiency *Sp* of cells larger than 1 μm is calculated as follows:

10 $${Sp}=\left(1-\frac{{N}_{p}}{{N}_{W}}\right)\times 100 \%$$

Where *N*_*p*_ and *N*_*W*_ present the number of cells larger than 1 μm in plasma and whole blood, respectively. Particle counting was conducted using flow cytometry.

The plasma recovery rate *R* is determined using the equation as follows:

11 $$R=\frac{{V}_{p}}{{V}_{W}\times \left(1-{hct}\right)}\times 100 \%$$

Where *V*_*p*_ and *V*_*W*_ represent the volumes of plasma and whole blood, respectively, and *hct* denotes hematocrit.

### Flow cytometry measurements

Prior to sample measurements, the flow cytometer (FCM, CytoFLEX S, Backman Coulter) was stabilized by running filtered (0.22 μm) ddH_2_O for at least 30 min to minimize instrument background noise. Calibration was performed using a set of reference polystyrene beads, including 0.5 μm, 1 μm and 2 μm beads (Thermo Fisher Scientific, USA), respectively. Following system calibration, whole blood samples were diluted 1000-fold while plasma samples were diluted 100-fold with PBS buffer, all samples were analyzed using the FSC and SSC channels.

### Hemolysis assay

Hemolysis was quantified spectrophotometrically by measuring the optical density (OD) at wavelengths of 540 nm and 576 nm, which correspond to free hemoglobin concentrations in plasma samples. To establish a positive control for whole blood lysis, samples were treated with 1% Triton X-100 (Sigma Aldrich, MO, USA) and then incubated at 37 °C for one hour, followed by centrifugation at ×1300 *g* for 10 min at 4 °C and ×4000 *g* for 15 min at 4 °C to obtain plasma solutions. A negative control was established using plasma solutions directly obtained from fresh whole blood via two-step centrifugation. OD measurements for all samples were conducted using a spectrophotometer (TGem Ultra, TIANGEN BIOTECH (BEIJING) CO., LTD).

### Plasma protein analysis

Plasma proteins obtained from magnetic separation and two-step centrifugation were isolated using a Plasma Protein Isolation Kit (Solarbio, Beijing, China), and the protein recovery rate was assessed using a BCA Protein Assay Kit (Epizyme, Shanghai, China). To verify the absence of non-specific protein depletion, proteins were separated by 12.5% SDS-PAGE and stained with Coomassie Brilliant Blue fast staining solution (Solarbio, Beijing, China) according to the manufacturer’s instructions. Gel imaging was performed using a gel imager (Analytik Jena, Langewiesen, Germany). A StarRuler Color Prestained Protein Marker (Genstar, Beijing, China) was utilized as a protein standard.

### Fluorescence correlation spectroscopy (FCS)

FCS was used to assess the concentration and diffusion dynamics of antibodies in plasma. Different concentrations of Alexa 647-conjugated antibodies were added to whole blood samples obtained from rats. Subsequently, the samples were processed using magnetic separation and two-step centrifugation to obtain the plasma.

FCS experiments were conducted using a commercial fluorescence correlation spectrometer (CorTector^TM^ SX200, LightEdge Technologies Limited, China) equipped with a 60 × 1.2NA UPLANSAPO water immersion objective (Olympus, Japan) and two continuous-wave solid-state lasers (488 nm, 638 nm; Pavilion Integration Corporation, China). The FCS instrument is based on a confocal optical pathway, using a multimode optical fiber with an inner core diameter of 50 μm as the confocal pinhole. The 638 nm laser was used for excitation, and emission light in the spectral range of 650–900 nm was detected using an optical fiber-coupled single-photon counting avalanche photodiode detector (SPCM-800-14-FC, Excelitas Technologies Corporation, USA). The excitation power was set at 5 mW output, with an actual laser power of 12.8 μW measured after the microscope objective. Prior to each experiment session, the confocal pinhole was aligned using a 10 nM ATTO655 carboxyl acid (ATTO-TEC GmbH, Germany) solution. FCS data from the calibration sample was collected and analyzed using the *Correlation Acquisition* and *Correlation Analysis* software (LightEdge Technologies Limited, China), respectively. The known diffusion coefficient of the ATTO655 dye (4.26 × 10^6 ^cm^2 ^s^-1^) was utilized to determine the structure factor (*S*) and confocal volume (*V*) of the FCS instrument, which were subsequently used for sample data analysis. Experimental temperatures were recorded and utilized to automatically correct for temperature-induced changes in molecular diffusion during data analysis. For FCS experiments using plasma containing Alexa 647-conjugated IgG (Abcam, UK), approximately ~30 μL of the solution was placed on a high-quality glass coverslip (CG15CH/CG15KH; Thorlabs, USA) positioned directly above the objective lens. Autocorrelation curves were recorded for 10 s and repeated 10 times for each sample.

### SARS-CoV-2 S-ECD Trimer IgG binding assay

The serial dilution curves for the S-ECD IgG binding assays were obtained using house-developed tip optofluidic immunoassay (TOI). Detailed description of the immunoassay platform and assay methodology can be found in our previous publication^44^. In this set of demonstration-of-concept experiments, recombinant trimeric SARS-CoV-2 S-ECD protein (wild type) was coated on the inner surface of the microfluidic immune-reactors at 5 μg/mL. 1% casein in PBS was used as the blocking and sample/reagent dilution buffer.

## Supplementary information

Supporting Information

Movie S1 Rat whole blood separation: indicating the relationship between rat blood cell separation time and separation distance

Movie S2 Human whole blood separation: indicating the relationship between human blood cell separation time and separation distance
