# Supplementary material for: Power-free plasma separation based on negative magnetophoresis for rapid biochemical analysis
Source: Microsyst Nanoeng. 2024 Dec 31;10:207. doi: 10.1038/s41378-024-00837-8 (PMC11688449; doi:10.1038/s41378-024-00837-8)
Supplement: Supplementary file 1 — Supporting Information [file 41378_2024_837_MOESM1_ESM.docx]

Power-Free Plasma Separation Based on Negative Magnetophoresis for Rapid Biochemical Analysis

Lin Zeng^a^, Chao Liu^a,b^, Yi Yang^a,b^, Shi Hu^a^, Ruihan Li^a^, Xiaotian Tan^a^, Jienan Shen^a^, Yi Zhang^c^, Shaohui Huang^d^, and Hui Yang^a,^*

a Research Center for Bionic Sensing and Intelligence, Institute of Biomedical and Health Engineering, Shenzhen Institute of Advanced Technology, Chinese Academy of Sciences, 518055 Shenzhen, China.

b Marine Engineering College, Dalian Maritime University, 116026 Dalian, China.

c Research Center for Medical AI, Institute of Biomedical and Health Engineering, Shenzhen Institute of Advanced Technology, Chinese Academy of Sciences, 518055 Shenzhen, China.

d School of Biosciences, University of Chinese Academy of Sciences, 101408 Beijing, China.

* Corresponding Author. E-mail: hui.yang@siat.ac.cn.

This PDF file includes:

Fig. S1 Structure design of the separation channel

Fig. S2 Separation channels for different throughputs

Fig. S3 The concentration distribution of ferrofluid along the y-axis

Fig. S4 Photographs of high-throughput plasma separation using a 3 mL/cycle channel

Fig. S5 SDS-PAGE analysis of human plasma obtained by separation system and centrifugation

Fig. S6 Flow cytometry results of 5 human blood samples: whole blood, human plasma obtained by separation system and centrifugation

Fig. S7 Microscope photos the human whole blood and the plasma obtained by the magnetic separation system and centrifugation

Fig. S8 Comparison of the separation efficiency of 5 human blood samples using magnetic separation and centrifugation

Fig. S9 SARS-CoV-2 S-ECD trimer IgG titration curve of the human plasma (S2-S5) obtained by the magnetic separation system and centrifugation

Fig. S10 Separation performance summary: the device can adapt to the whole blood separation of all volumes in all scenarios, and its adaptability and separation efficiency far exceed the current centrifuge and microfluidic methods.

Fig. S11 A) Method for removing ferrofluid from plasma after magnetic separation; B) Comparison of ferrofluid removal effects; C) Comparison of spectral results between the magnetically separated plasma after removal of ferrofluid and the centrifuged plasma

Tab. S1 Comparison of plasma separation methods

Tab. S2 Parameters used in the simulations

Movie S1 Rat whole blood separation: indicating the relationship between rat blood cell separation time and separation distance

Movie S2 Human whole blood separation: indicating the relationship between human blood cell separation time and separation distance


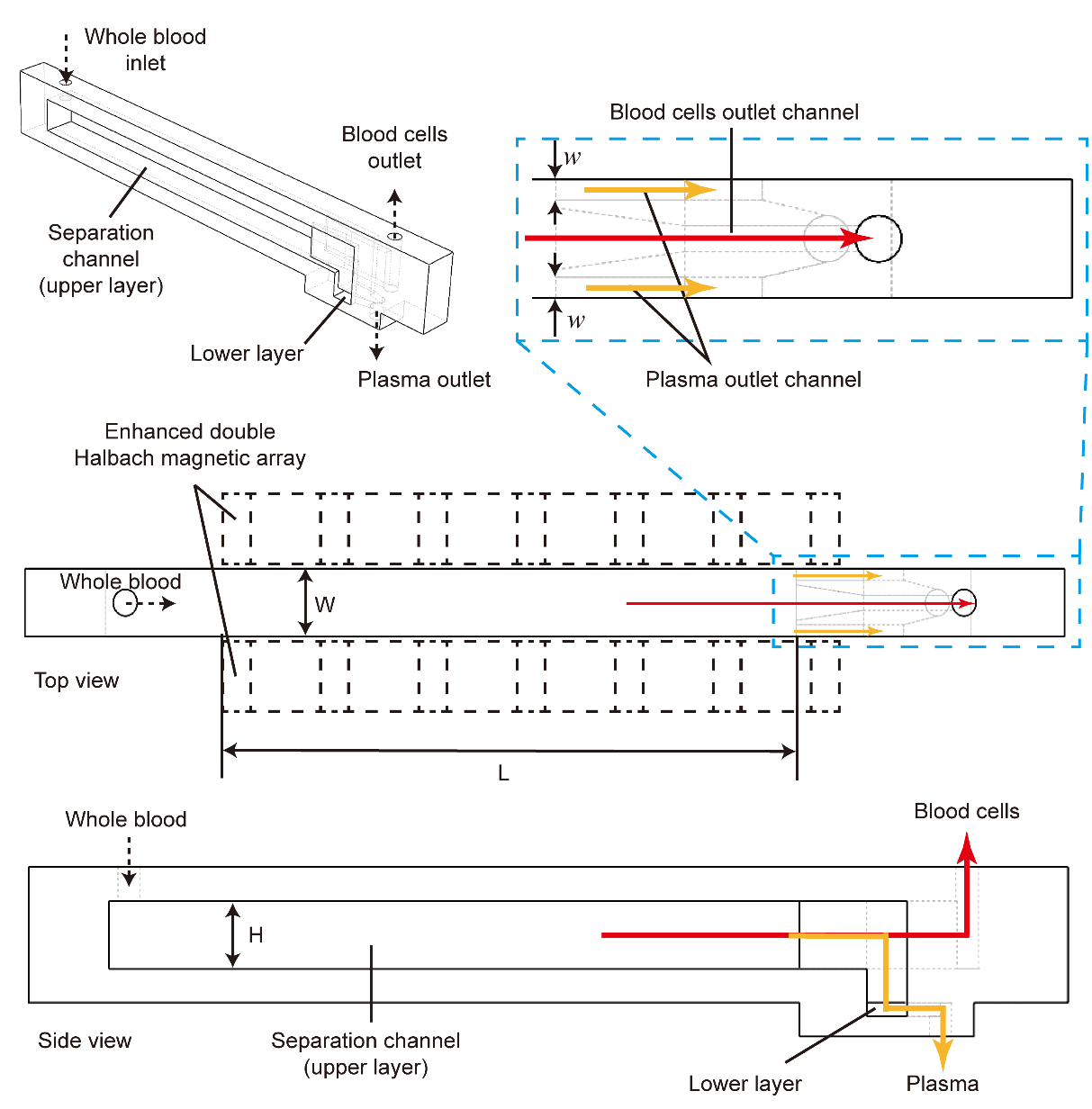


Fig. S1 Structure design of the separation channel


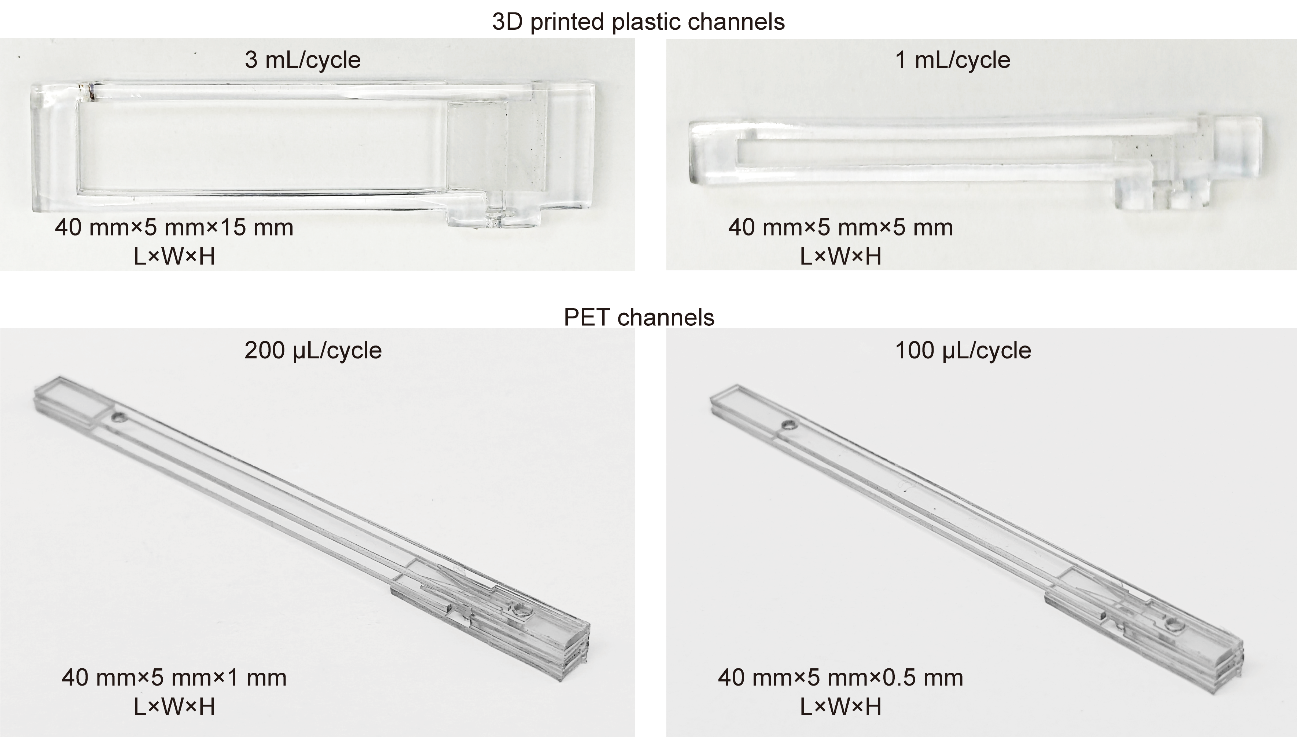


Fig. S2 Separation channels for different throughputs


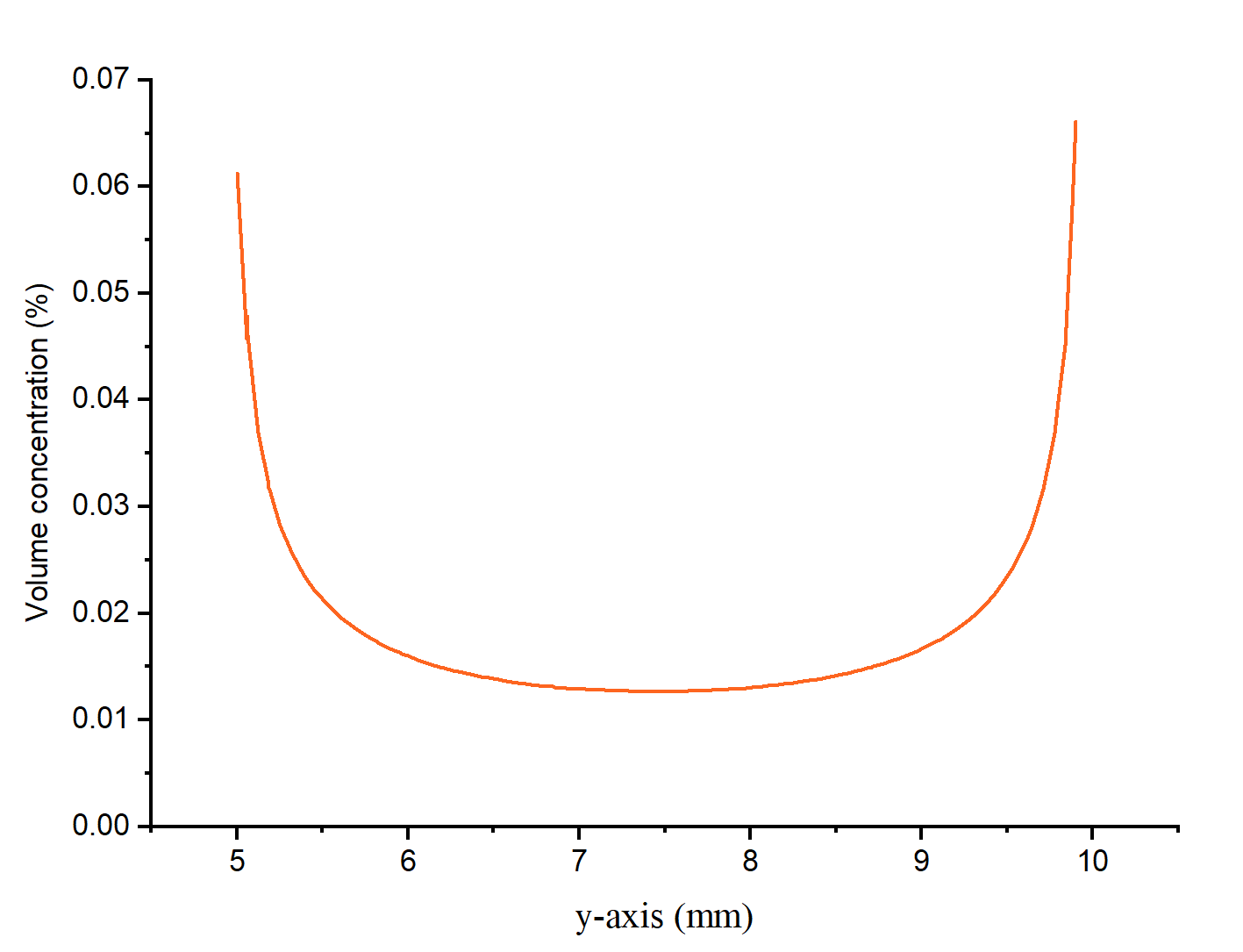


Fig. S3 The concentration distribution of ferrofluid along the y-axis


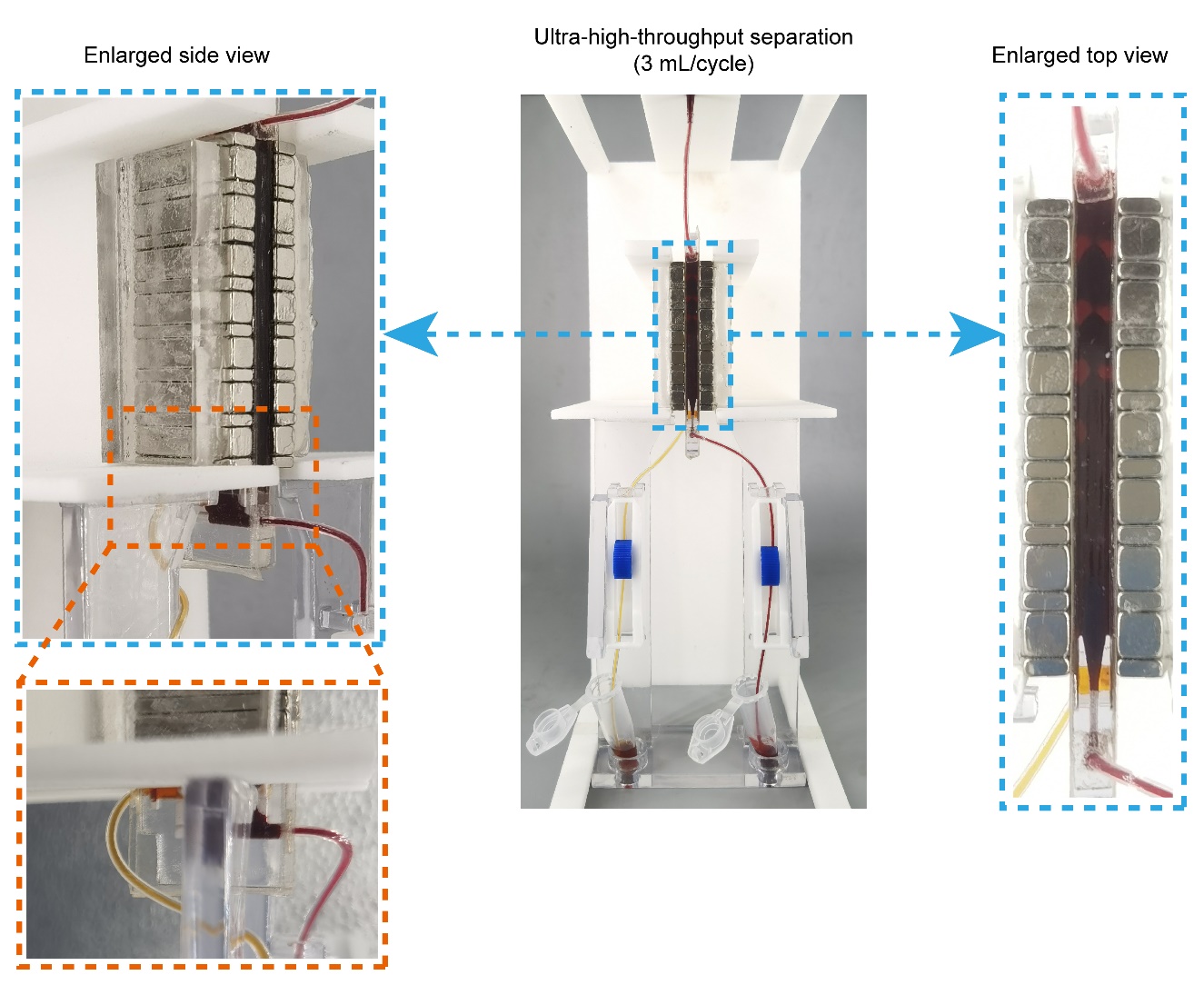


Fig. S4 Photographs of high-throughput plasma separation using a 3 mL/cycle channel


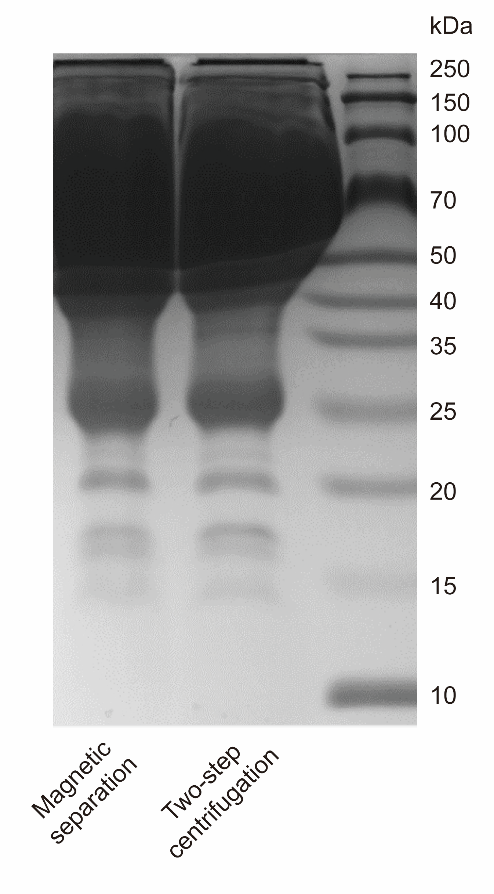


Fig. S5 SDS-PAGE analysis of human plasma obtained by separation system and centrifugation


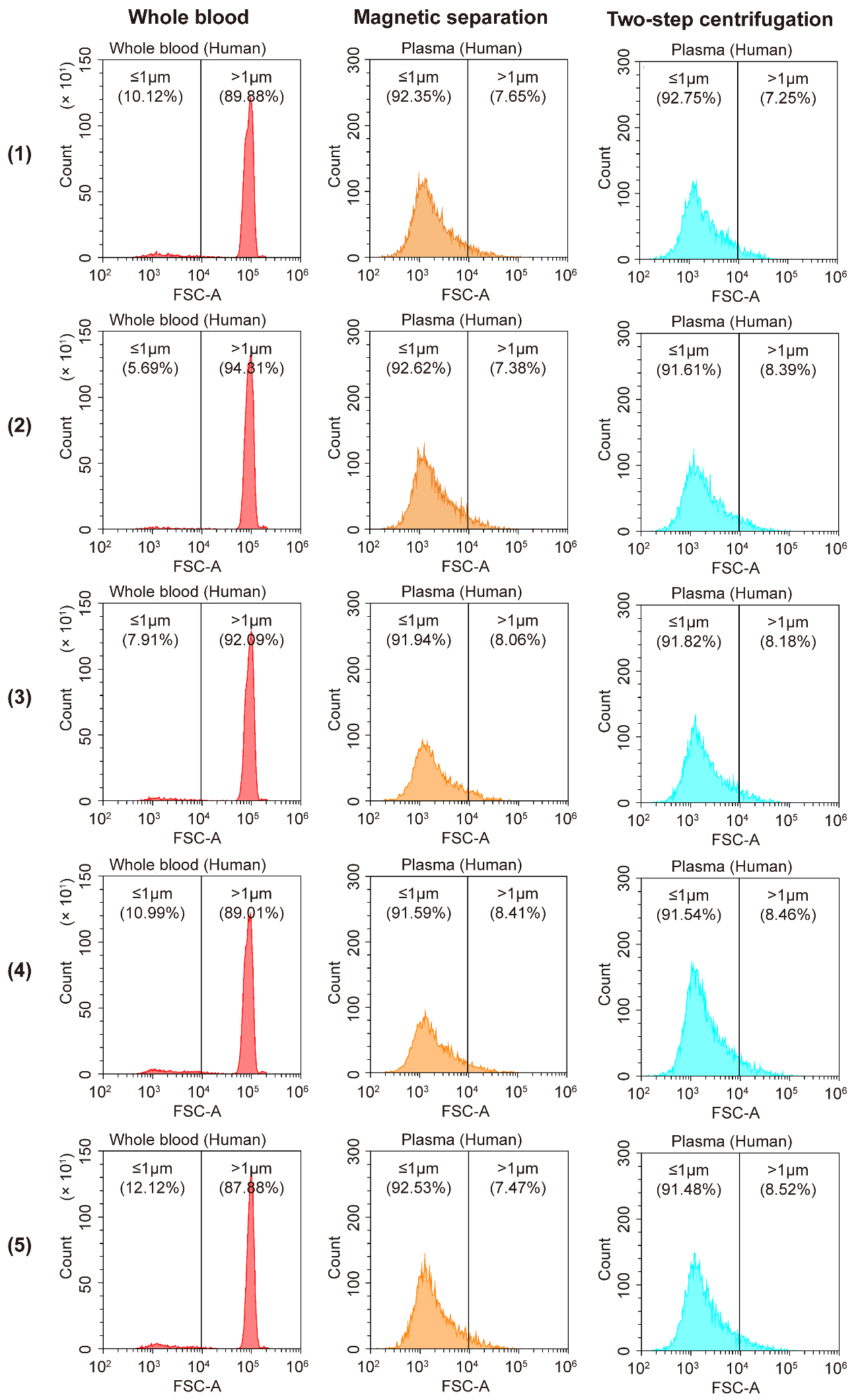


Fig. S6 Flow cytometry results of 5 human blood samples: whole blood, human plasma obtained by separation system and centrifugation
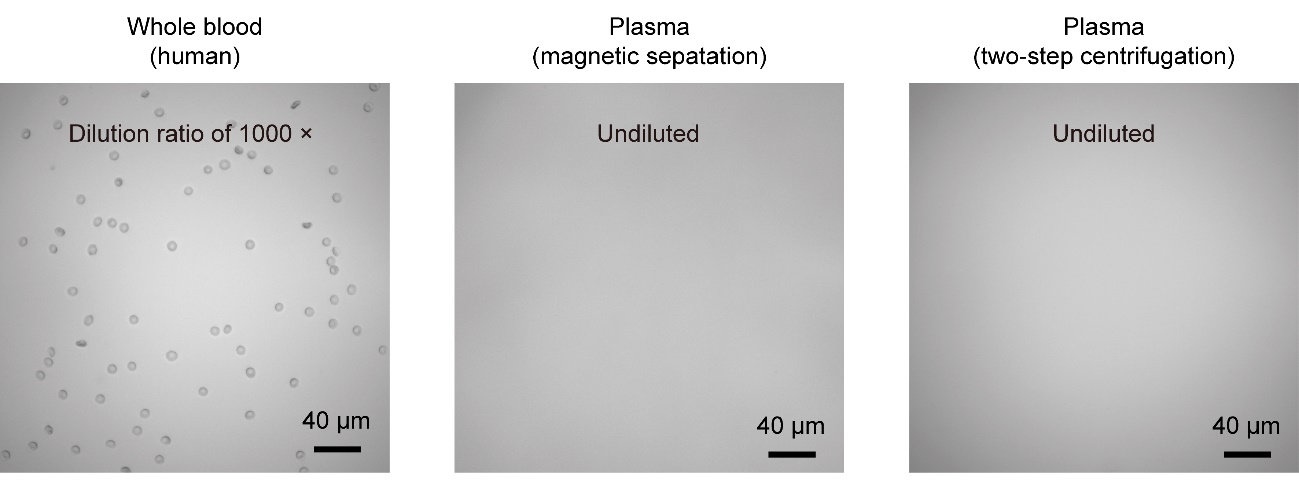


Fig. S7 Microscope photos the human whole blood and the plasma obtained by the magnetic separation system and centrifugation


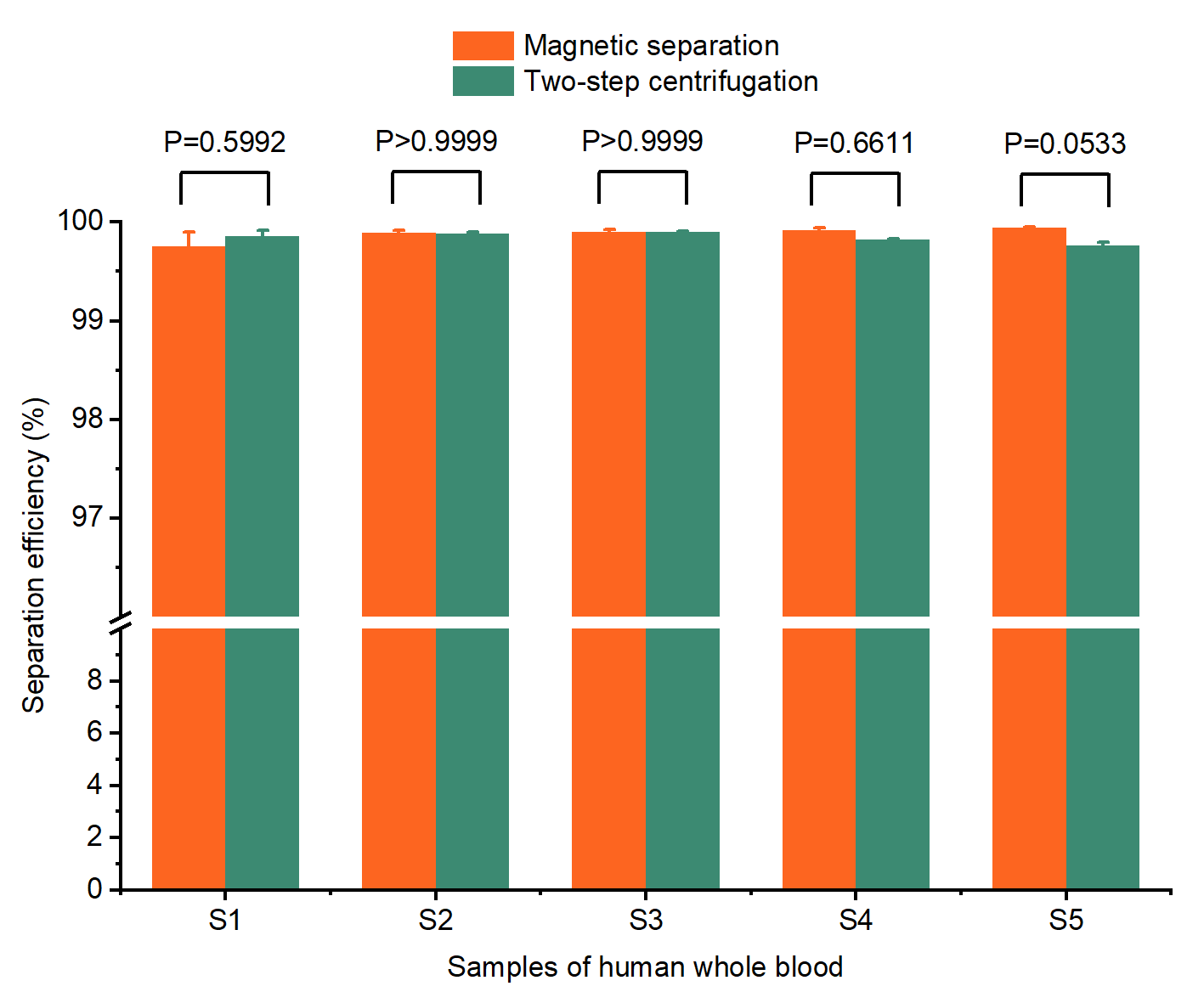


Fig. S8 Comparison of the separation efficiency of 5 human blood samples using magnetic separation and centrifugation


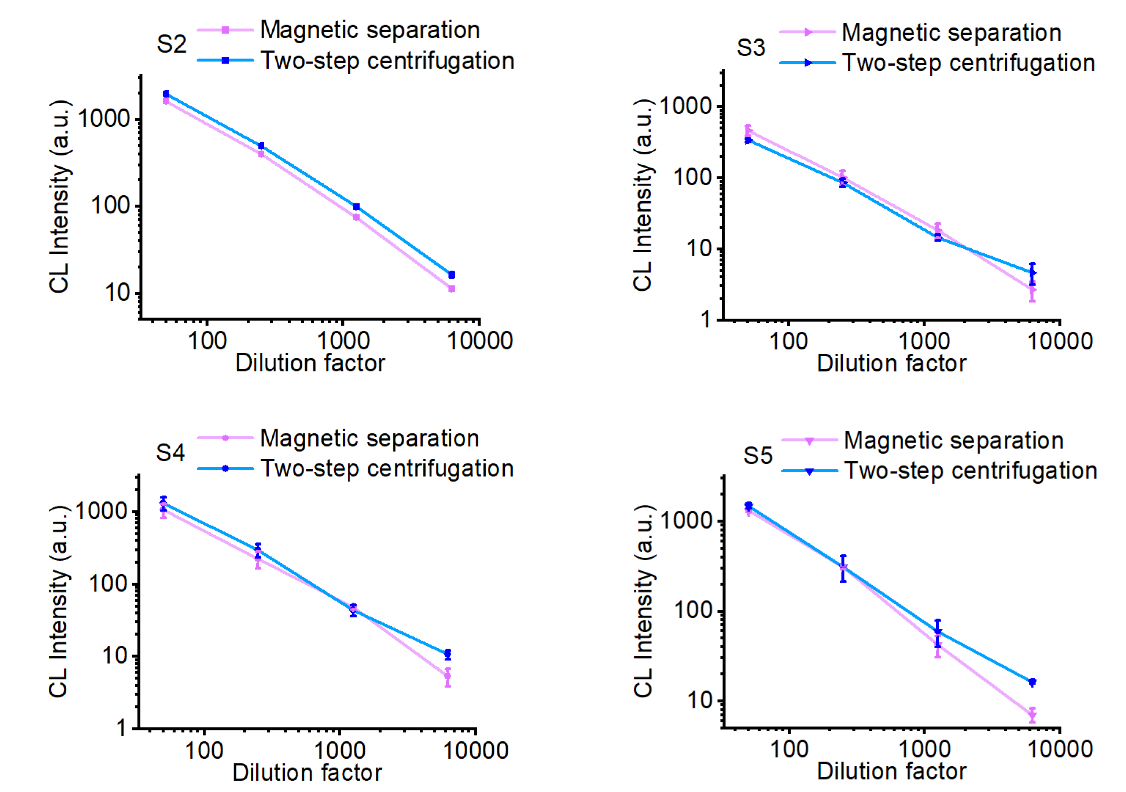


Fig. S9 SARS-CoV-2 S-ECD trimer IgG titration curve of the human plasma (S2-S5) obtained by the magnetic separation system and centrifugation


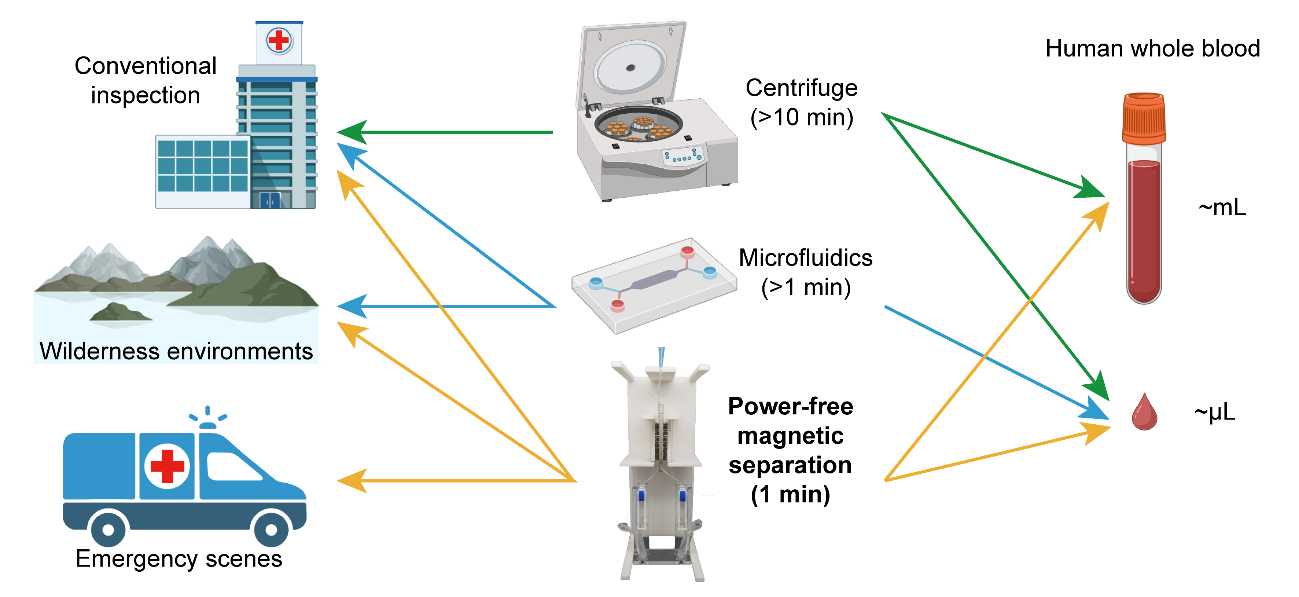


Fig. S10 Separation performance summary: the device can adapt to the whole blood separation of all volumes in all scenarios, and its adaptability and separation efficiency far exceed the current centrifuge and microfluidic methods


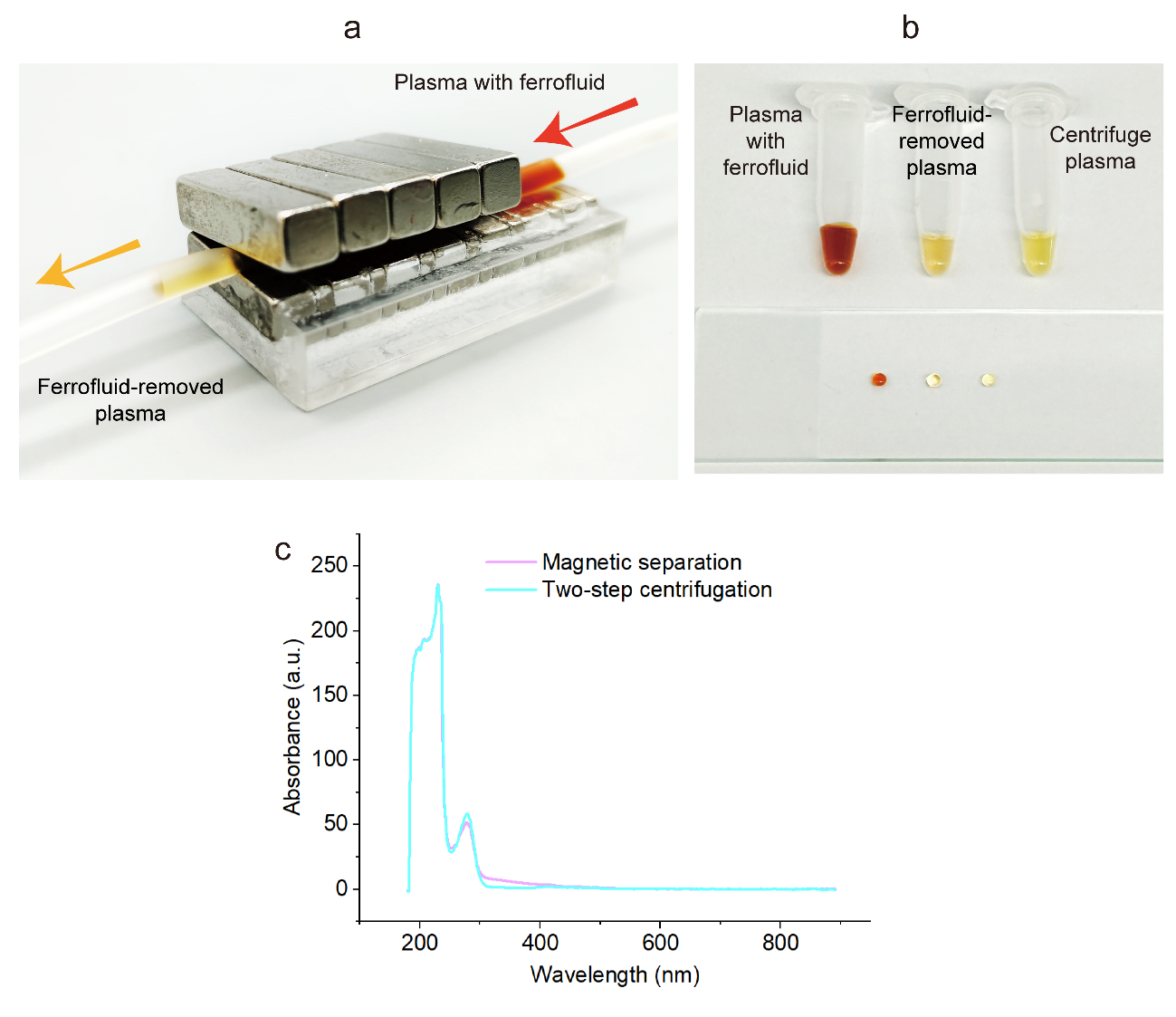


Fig. S11 **a** Method for removing ferrofluid from plasma after magnetic separation; **b** Comparison of ferrofluid removal effects; **c** Comparison of spectral results between the magnetically separated plasma after removal of ferrofluid and the centrifuged plasma

Tab. S1 Comparison of plasma separation methods

| **Methods** | **Sample** | **Throughput** | **Plasma recovery volume/recovery rate (hct=45%)** | **Blood cell removal rate** | **Whole blood separation rate** | **Power-free device** |
| --- | --- | --- | --- | --- | --- | --- |
| Magnetophoresis [19] | Whole blood | 1000 μL | 420 μL/76% | - | 1300 μL /min | Yes |
| Negative magnetophoresis [20] | Whole blood | 4000 μL | 1832.6 μL/83.3% | 100% | 100 μL/min | No |
| Dielectric forces [18] | Whole blood | 5 μL | 2 μL/72.7% | red blood cells: 98.7% | 1.25 μL/min | No |
| Electrophoretic force and gravity effects [17] | Whole blood | 10 μL | 2.2μL/40% | red blood cells: 99.98% | 4.2-6 μL/min | No |
| Sound waves [15] | Whole blood | 50 μL | 15.9μL/57.8% | red blood cells: 99.9% | 5.8 μL/min | No |
| Microfluidics-based membrane filtration [22] | Whole blood | 100 μL | 12 μL/21.8% | - | 10 μL /min | No |
| Microfluidics-based Membrane filtration [23] | Whole blood | 800 μL | 280 μL/63.6% | red blood cells: 99.9%;  white blood cells: 96.9% | 160 μL /min | Yes |
| Microfluidics-based membrane filtration [26] | Whole blood | 160 μL | 22 μL/25% | - | 10.67 μL /min | Yes |
| Conventional membrane filtration [21] | Diluted whole blood | 1000 μL (including 10 μL whole blood) | - | - | 20 μL /min | Yes |
| Membrane filtration with capillary effects [25] | Whole blood | 60 μL | 23.7 μL/71.7% | red blood cells: 99.8%;  white blood cells: 91.8% | 10 μL/min | Yes |
| Fluid dynamics [27] | Diluted whole blood | 24 mL (including 265-530 μL whole blood) | - | Almost 100% | 530 μL /min | No |
| Fluid dynamics [28] | Whole blood | 500 μL | 30 μL/10.9% | 99.5% | 500 μL /min | No |
| Fluid dynamics [29] | Whole blood | 2000 μL | 242 μL/22% | red blood cells: 97% | 33.3 μL /min | No |
| Fluid dynamics [30] | Whole blood | 4000 μL | 340 μL/15.5% | red blood cells: 100% | 66.7 μL/min | No |
| Centrifugal-based fidget-spinner [31] | Whole blood | 10 μL | 3 μL/54.5% | 99% | 2.5 μL /min | Yes |
| Paper centrifuge [32] | Whole blood | 20 μL | 8 μL/70.2%  (hct=43%) | 100% | 13.3 μL /min | Yes |
| **Negative magnetophoresis**  **(This paper)** | **Whole blood** | **100-3000 μL** | **40-1200 μL /72.7%** | **99.9%** | **3000 μL/min**  **(Human whole blood)** | **Yes** |

References in manuscript.

Tab. S2 Parameters used in the simulations

| Parameters | Value |
| --- | --- |
| Remanent flus density of the magnets | 1.48 T |
| Saturation magnetization of ferrofluid | 25.2 Am^2^/kg |
| Dynamic viscosity of the sample | 4 mPa•s |

Movie S1 Rat whole blood separation: indicating the relationship between rat blood cell separation time and separation distance

Movie S2 Human whole blood separation: indicating the relationship between human blood cell separation time and separation distance
